# Dynamics of Citrate Synthase and Malate Dehydrogenase Gene Expression, Enzymatic Activity, and Physicochemical Changes During Postharvest Storage of Soursop (*Annona muricata* L.) GUANAY-1 Fruit

**DOI:** 10.3390/ijms27167174

**Published:** 2026-08-11

**Authors:** Vladimir Chavarin-Pérez, Lilia Aurora Díaz-Rincón, José Orlando Jiménez-Zurita, Graciela Guadalupe López-Guzmán, Gladys Alejandra Toledo-Ibarra, Cindy Sacnithe Agredano-De La Garza, Verónica Alhelí Ochoa-Jiménez, Guillermo Berumen-Varela

**Affiliations:** 1 Programa de Maestría en Ciencias en Biotecnología, Universidad Autónoma de Nayarit, Carretera Tepic-Compostela, Km. 9, Xalisco 63780, Mexico; 24000297@uan.edu.mx; 2 Programa de Doctorado en Ciencias Biológico Agropecuarias, Universidad Autónoma de Nayarit, Carretera Tepic-Compostela, Km. 9, Xalisco 63780, Mexico; mc.lili.diaz@gmail.com; 3 Unidad Academica de Agricultura, Universidad Autónoma de Nayarit, Carretera Tepic-Compostela, Km. 9, Xalisco 63780, Mexico; jose-jimenez@uan.edu.mx (J.O.J.-Z.); graciela.lopez@uan.edu.mx (G.G.L.-G.); 4 Laboratorio Nacional de Investigación para la Inocuidad Alimentaria (LANIIA)—Unidad Nayarit, Universidad Autónoma de Nayarit, Calle Tres S/N, Colonia, Cd. Industrial, Tepic 63000, Mexico; gladys.toledo@uan.edu.mx; 5 Estancias Posdoctorales por México, Secretaría de Ciencia, Humanidades, Tecnologia e Innovacion, Coordinacion de Apoyos a Becarios e Investigadores, Dirección de Posgrado, Ciudad de Mexico 03940, Mexico; cindyagredano@gmail.com (C.S.A.-D.L.G.); veronica.ochoa@uan.edu.mx (V.A.O.-J.); 6 Unidad de Tecnología de Alimentos-Secretaría de Investigación y Posgrado, Universidad Autónoma de Nayarit, Ciudad de la Cultura SN, Tepic 63000, Mexico

**Keywords:** enzyme, maturation, postharvest storage, transcriptional regulation, softening

## Abstract

Soursop (*Annona muricata* L.) is a climacteric tropical fruit belonging to the Annonaceae family, widely cultivated in tropical and subtropical regions due to its economic importance, nutritional value, and abundance of bioactive compounds. Its pulp is highly appreciated for fresh consumption and food processing; however, its high respiration rate and ethylene production accelerate ripening, resulting in rapid quality deterioration and a short postharvest shelf life. During ripening, soursop fruit undergoes physiological, biochemical, and molecular changes associated with organic acid metabolism, in which citrate synthase (CS) and malate dehydrogenase (MDH) play key roles within the tricarboxylic acid cycle. However, the transcriptional dynamics of genes associated with the tricarboxylic acid cycle during postharvest storage of tropical fruits remains poorly characterized. This study aimed to identify in silico genes encoding CS and MDH and to evaluate changes in physicochemical traits, enzymatic activity, and the expression of selected CS and MDH genes during postharvest storage of soursop (*A. muricata*) GUANAY-1 fruit. Fruits were stored at 26 ± 1 °C and evaluated on days 1, 2, 3, 4, and 5. Titratable acidity, pH, total soluble solids, CS activity, MDH activity, and the expression of selected CS and MDH genes were analyzed. Physicochemical analysis showed that the major changes in acidity, TSS, and pH occurred on day 3. In silico analysis identified 16 CS genes and 47 MDH genes in soursop, whereas transcriptome analysis identified four CS genes and three MDH genes with differential expression, designated as *CS1*, *CS2*, *CS3*, *CS4*, *MDH1*, *MDH2*, and *MDH3*, respectively. The Neighbor-Joining analysis grouped the CS sequences into two clusters and the MDH sequences into three clusters. Differential expression analysis revealed that *CS1*, *CS2*, *CS3*, *CS4*, *MDH1*, *MDH2*, and *MDH3* were upregulated on day 3 relative to day 0 of postharvest storage. Functional enrichment analysis indicated that these genes were mainly associated with cellular respiration and the tricarboxylic acid cycle. The highest CS activity was recorded on day 3, coinciding with the maximum expression of *CS1*, *CS2*, and *CS4*. In contrast, MDH activity peaked on day 2, whereas *MDH1* and *MDH2* reached their highest expression on day 3. Correlation analysis showed positive associations between *CS2* and *CS4* expression and both TSS and titratable acidity. Overall, these results demonstrate temporal changes in physicochemical traits, CS and MDH enzymatic activities, and the expression of selected *CS* and *MDH* genes during postharvest storage of GUANAY-1 soursop fruit.

## 1. Introduction

Soursop (*A. muricata*) is a tropical climacteric fruit belonging to the Annonaceae family and is characterized by its dark green peel covered with soft fleshy spines [1]. In 2025, Mexico produced approximately 30,000 tons of soursop fruit [2], of which nearly 24,700 tons were harvested in the state of Nayarit, making it the country’s leading producer. Three soursop cultivars developed in Nayarit—GUANAY-1, GUANAY-2, and GUANAY-3—have been officially registered in the Mexican National Catalogue of Plant Varieties, showing different morphological characteristics [3]. Among them, GUANAY-1 exhibits desirable agronomic and commercial characteristics, including heart-shaped fruits with a rounded apex, an average weight of approximately 1.4 kg, and small (0.5 mm), densely distributed fleshy spines on the peel.

Previous postharvest studies have shown that storage at 15 °C extends the shelf life of GUANAY-1 fruits by approximately three days compared with storage at 28 °C, highlighting its potential for long-distance transportation and commercialization in domestic and international markets [4]. Despite these favorable characteristics, GUANAY-1, like other soursop cultivars, exhibits a relatively short postharvest life because of its climacteric nature.

Climacteric fruits undergo a marked increase in respiration and ethylene production during ripening, processes that regulate numerous physiological and biochemical changes associated with fruit quality and senescence [5,6]. In soursop, these events promote rapid postharvest ripening, which is characterized by changes in peel color, aroma, flavor, and texture as the fruit progresses from physiological maturity to consumption ripening and finally softening [6,7,8]. During this process, sugar accumulation occurs simultaneously with changes in pH and organic acid content, resulting in the characteristic sweet–acid flavor of ripe soursop fruit [9]. Sugars and organic acids are the major soluble compounds determining fruit flavor and overall quality. Whereas soluble sugars contribute to sweetness, organic acids determine acidity and are synthesized from transported carbohydrates, making the balance between both components essential for fruit quality during ripening [10].

**Figure 1:**
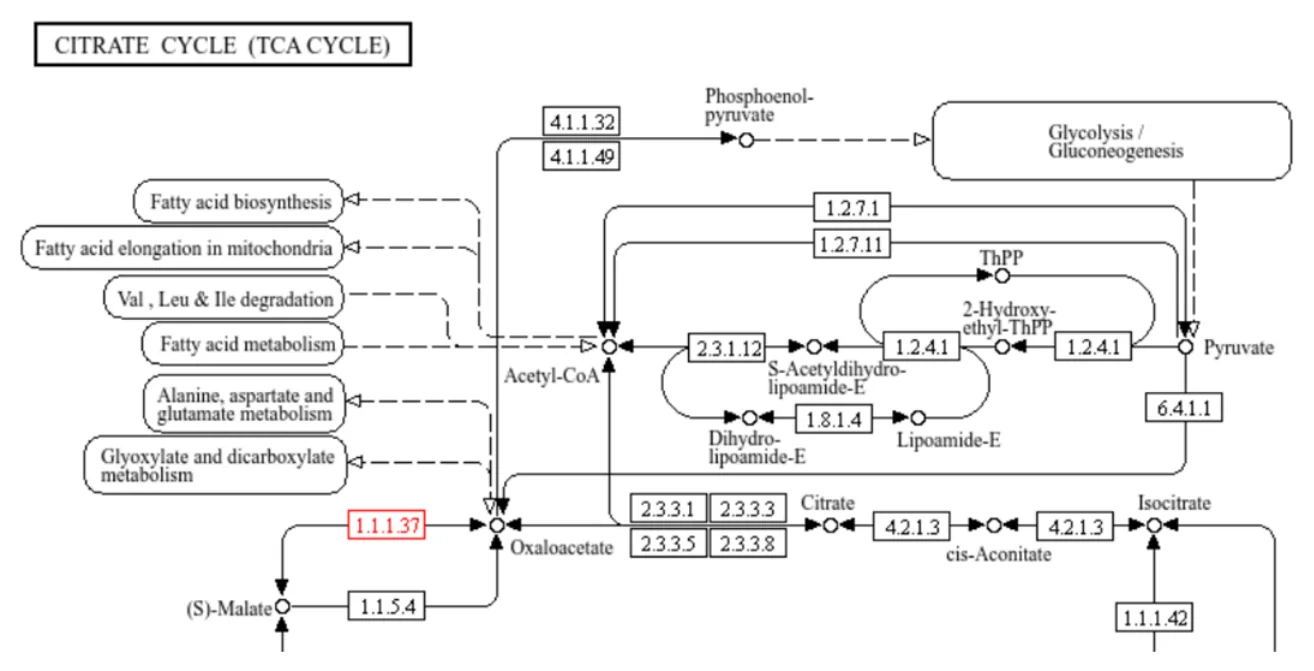
Simplified representation of the TCA cycle highlighting the reactions catalyzed by citrate synthase (CS) and malate dehydrogenase (MDH). Citrate synthase (EC 2.3.3.1) catalyzes the condensation of oxaloacetate and acetyl-CoA to form citrate, whereas malate dehydrogenase (EC 1.1.1.37) catalyzes the reversible conversion of malate to oxaloacetate. The pathway was adapted from the Kyoto Encyclopedia of Genes and Genomes (KEGG) database.

Within the tricarboxylic acid (TCA) cycle, sucrose-derived metabolites are converted into malate through glycolysis, whereas citrate synthase (CS) catalyzes the condensation of acetyl-CoA and oxaloacetate to produce citrate (Figure 1), representing the first irreversible step of the TCA cycle [11,12,13]. According to Etienne et al. [12]., citric acid accumulates in the mitochondria through the TCA cycle, in which CS catalyzes the formation of citrate. In contrast, malate predominantly accumulates in the vacuole and its metabolism is closely associated with malate dehydrogenase (MDH), which catalyzes the reversible conversion between oxaloacetate and malate (Figure 1). Malate may also be synthesized from phosphoenolpyruvate generated during glycolysis through the action of phosphoenolpyruvate carboxylase (PEPC).

Beyond their contribution to fruit acidity, organic acids are essential components of fruit metabolism and play critical roles during ripening. Among them, citrate and malate participate as central intermediates of the TCA cycle while also contributing to carbon storage, energy metabolism, and the maintenance of cellular redox balance.

The concentrations of these organic acids are continuously adjusted through coordinated metabolic and transcriptional regulation in response to the physiological and biochemical changes that occur during ripening. Consequently, investigating the mechanisms controlling citrate and malate metabolism is fundamental to understanding the regulation of fruit quality attributes during postharvest ripening [12,14].

Nevertheless, soursop exhibits an atypical ripening pattern characterized by the accumulation of organic acids during postharvest ripening. Palomino-Hermosillo et al. [15] performed RNA-seq analysis on GUANAY-1 soursop fruits stored at 28 °C, revealing changes in the expression of genes involved in sugar metabolism and organic acid biosynthesis, a process that impacts fruit quality attributes, including flavor and firmness. Likewise, Ochoa-Jiménez et al. [16] carried out an untargeted metabolomic profiling using ultra-performance liquid chromatography coupled with quadrupole time-of-flight mass spectrometry (UPLC-QTOF-MS) on GUANAY-1 soursop fruits. A total of 68 metabolites were identified, among which citric acid and malic acid were the predominant organic acids throughout fruit development. More recently, the integration of transcriptomic and metabolomic datasets revealed coordinated interactions between genes and metabolites involved in sugar metabolism and organic acid biosynthesis, with the highest metabolic activity occurring during physiological maturity (day zero) and consumption ripening (day three) [17].

Although previous transcriptomic and metabolomic studies have considerably improved the understanding of soursop ripening, they primarily described global changes in gene expression and metabolite accumulation without specifically evaluating candidate genes associated with organic acid metabolism at both the transcriptional and enzymatic levels. In particular, these studies did not comprehensively characterize the CS and malate MDH gene families, quantify the relative expression of individual isoenzymes during postharvest storage by quantitative real-time PCR (qRT-PCR), determine the enzymatic activities of CS and MDH, or relate these molecular responses to physicochemical changes occurring during fruit ripening. Therefore, the present study integrates transcriptomic information, gene expression analysis, enzymatic activity measurements, and physicochemical characterization to provide a comprehensive temporal description of these processes during postharvest storage.

Such information contributes to a better understanding of the physiological and molecular changes associated with soursop ripening and supports the exploitation of the commercial potential of the GUANAY-1 cultivar, which is distinguished by its favorable physicochemical characteristics and extended postharvest performance. To the best of our knowledge, this is the first study to combine the in silico identification of CS and MDH genes with gene expression analysis, enzymatic activity measurements, and physicochemical characterization during postharvest ripening of soursop fruit.

Therefore, the objective of this study was to identify in silico genes encoding CS and MDH and to evaluate changes in physicochemical traits, enzymatic activity, and the expression of selected CS and MDH genes during postharvest storage of soursop (*A. muricata*) GUANAY-1 fruit.

## 2. Results

### 2.1. Physicochemical Analysis

**Figure 2:**
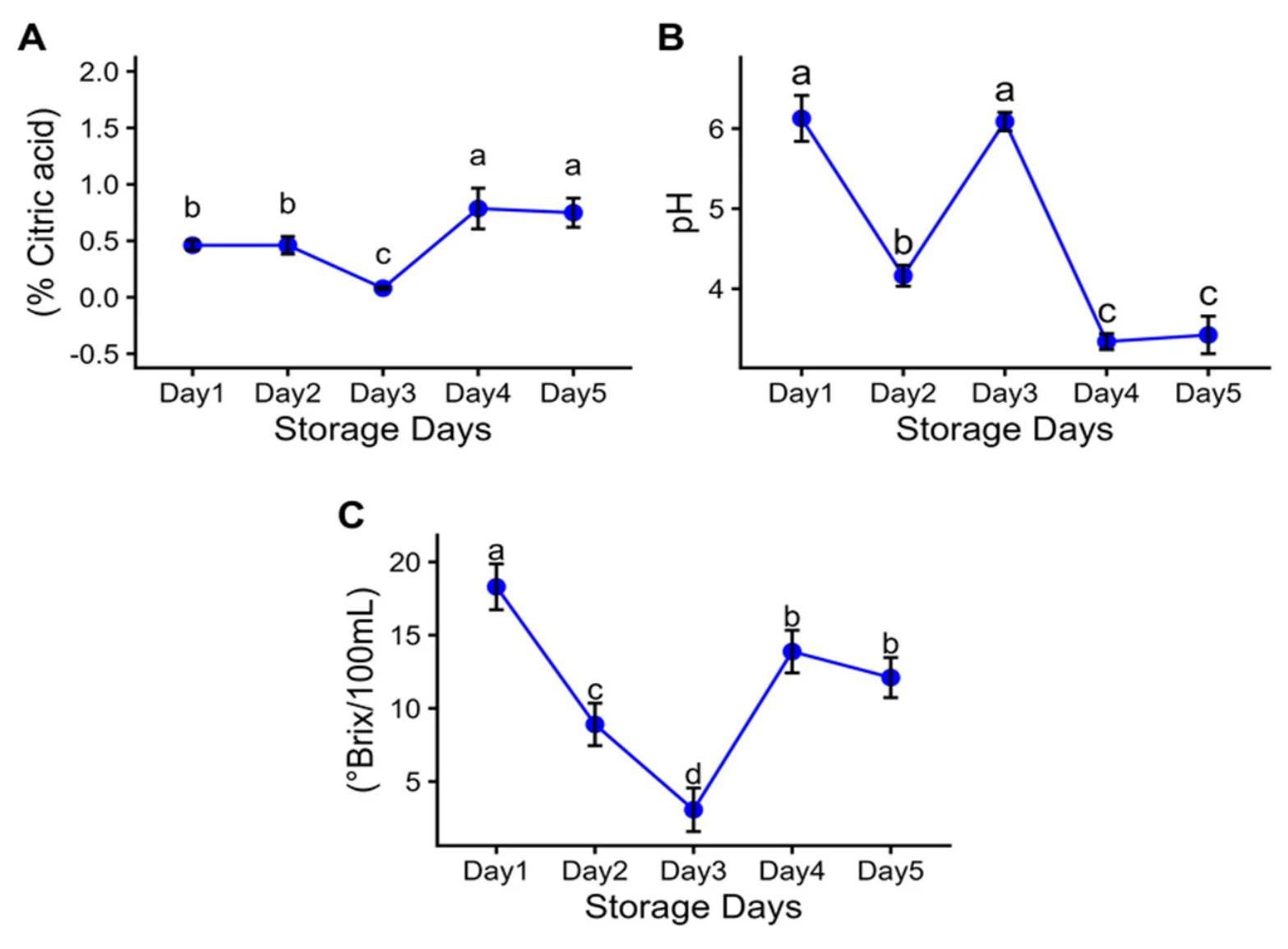
Changes in titratable acidity (**A**), pH (**B**), and total soluble solids (**C**) in GUANAY-1 soursop fruits during storage at 26 ± 1 °C. Different letters indicate significant differences among storage days according to Tukey’s HSD test (*p* < 0.05). Vertical bars represent the standard error.

Figure 2 shows the changes in titratable acidity, pH, and total soluble solids (TSS) in GUANAY-1 soursop fruits. Regarding titratable acidity (Figure 2A), values of 0.47% and 0.46% citric acid were recorded on days 1 and 2 of storage, respectively (*p* > 0.05). However, acidity significantly decreased to 0.08% on day 3 (*p* < 0.05), while on days 4 and 5 it increased to 0.79% and 0.75%, respectively (*p* > 0.05). On the other hand, pH showed significant variations during storage (Figure 2B). Between days 1 and 2, a decrease from 6.12 to 4.16 was observed (*p* < 0.05). Subsequently, on day 3, pH increased to 6.08, reaching values similar to those recorded at the beginning of storage and differing significantly from those observed on days 2, 4, and 5 (*p* < 0.05). During days 4 and 5, the lowest pH values were recorded (3.34 and 3.42, respectively), with no significant differences between them (*p* > 0.05). Regarding TSS (Figure 2C), a progressive decrease was observed during the first three days of storage, with values decreasing from 18.3 °Brix on day 1 to 8.9 and 3.0 °Brix on days 2 and 3, respectively, showing statistically significant differences among these days (*p* < 0.05). Subsequently, TSS increased to 13.8 °Brix on day 4 and remained at 12.1 °Brix on day 5 (*p* > 0.05).

### 2.2. Enzymatic Activity

**Figure 3:**
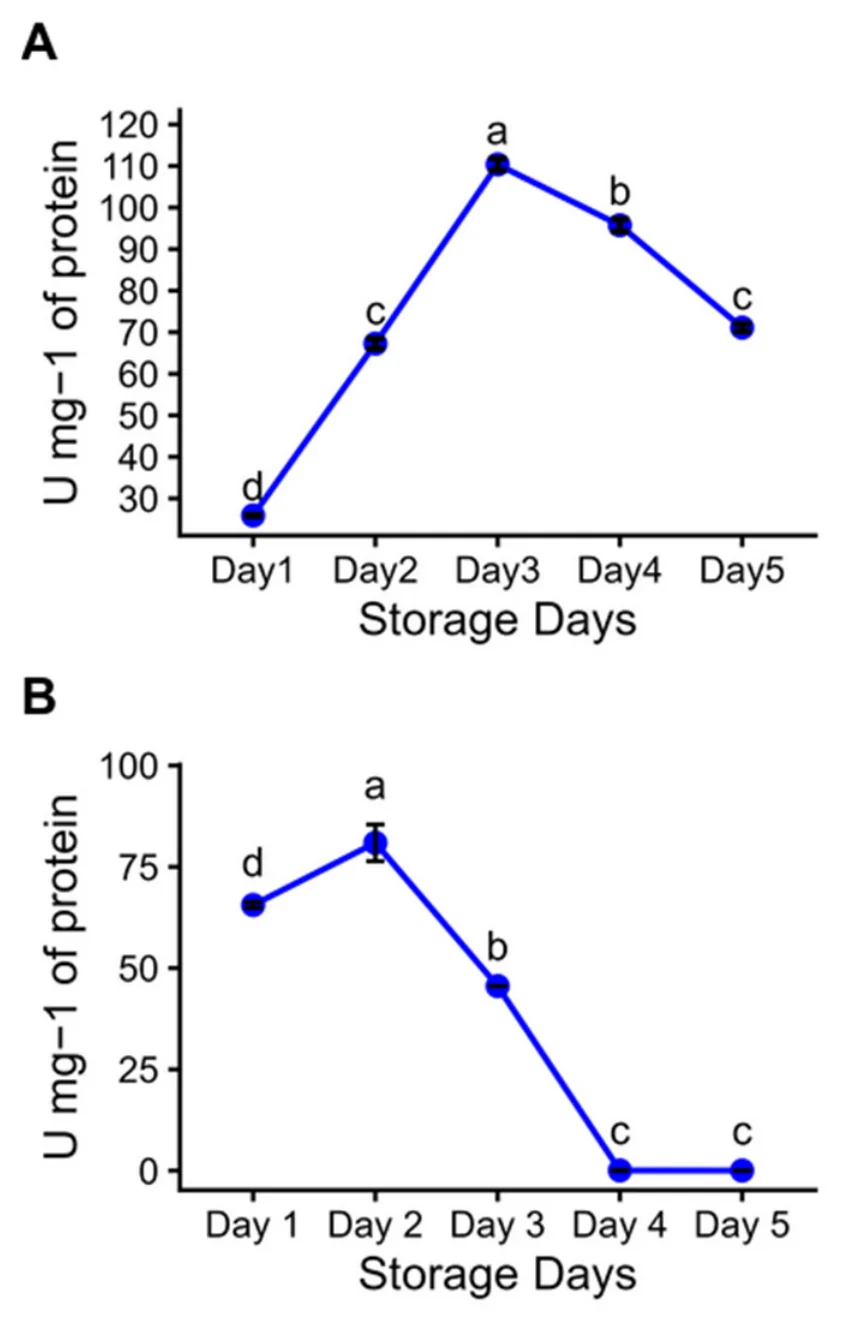
Activity of CS (**A**) and MDH (**B**) enzymes in GUANAY-1 soursop fruits during storage at 26 ± 1 °C. The *x*-axis shows storage days, and the *y*-axis shows enzymatic activity expressed as U mg^−1^ protein. Different letters indicate significant differences among storage days according to Tukey’s HSD test (*p* < 0.05). Bars represent the standard deviation of the means.

The enzymatic activities of CS and MDH were determined, showing significant differences among storage days for both enzymes (*p* < 0.05), as shown in Figure 3. The highest CS activity was observed on day 3 (110.36 U mg^−1^ protein), followed by a significant decrease on day 4 (95.73 U mg^−1^ protein; *p* < 0.05) and on day 5 (71.10 U mg^−1^ protein), as shown in Figure 3A. Regarding MDH activity (Figure 3B), a peak was observed on day 2 of storage (80.92 U mg^−1^ protein). A subsequent decrease was recorded on day 3, with values of 45.57 U mg^−1^ protein. No enzyme activity was detected on days 4 and 5 (*p* > 0.05).

### 2.3. Bioinformatic Analysis

#### 2.3.1. Phylogenetic Analysis of CS

**Figure 4:**
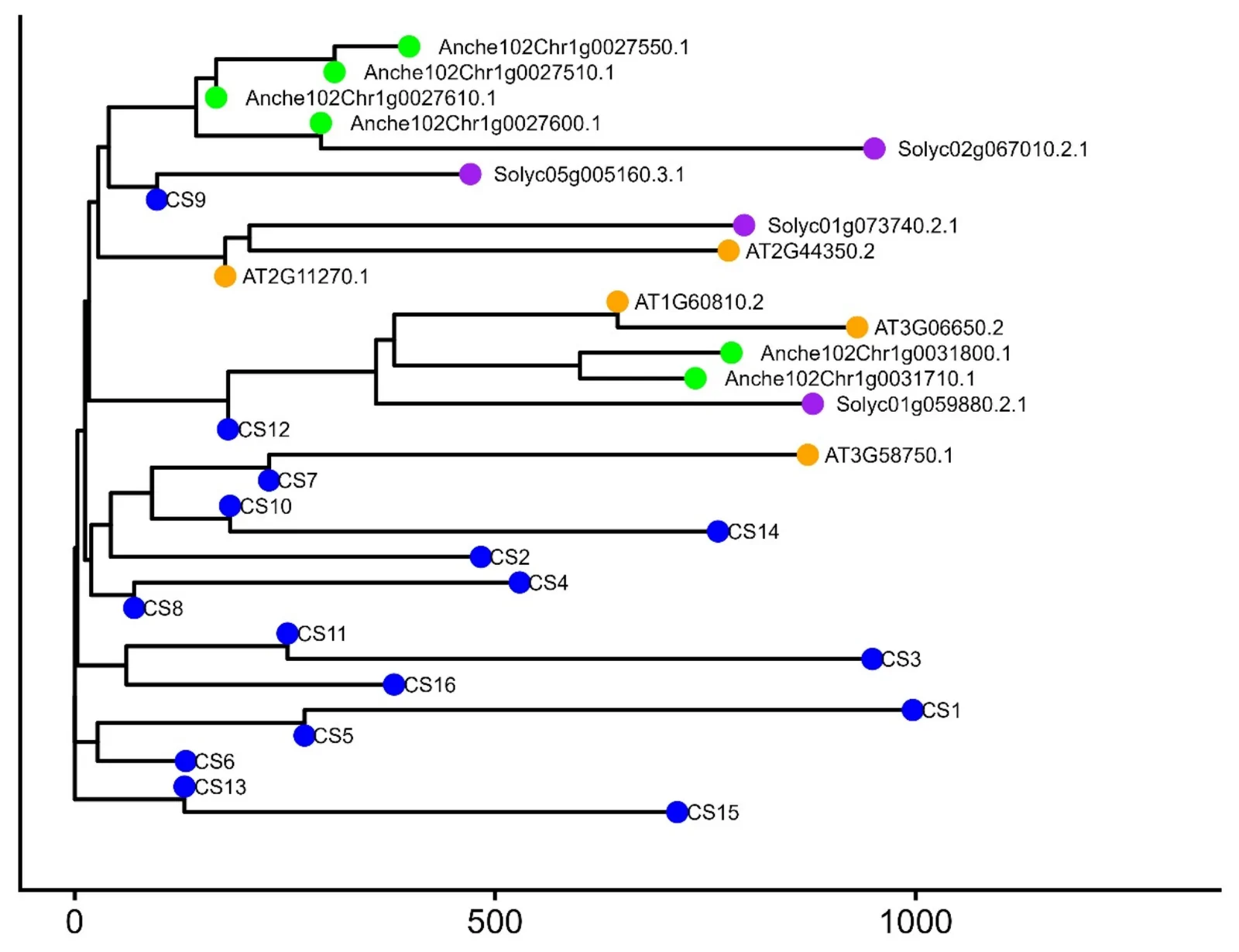
Neighbor-Joining phylogenetic tree of genes encoding the CS enzyme. Blue corresponds to soursop, green to cherimoya, orange to *A. thaliana*, and purple to tomato. The values at the bottom (*x*-axis) represent the estimated genetic distance (branch length) derived from the multiple sequence alignment.

A total of 31 isoenzymes encoding CS were identified in soursop, cherimoya, tomato, and Arabidopsis, of which 16 corresponded to soursop fruits and contained the PF00285 protein domain (Figure 4). The Neighbor-Joining tree grouped most soursop isoenzymes within the same major cluster, although *CS1* and *CS3* were located on separate branches within this cluster. In contrast, *CS9* clustered together with homologous sequences from cherimoya and tomato. Likewise, *CS12* was grouped within a cluster containing two cherimoya and two *A. thaliana* sequences. Overall, the analyzed sequences from *A. thaliana* (orange), cherimoya (green), tomato (purple), and soursop (blue) were distributed among several clusters, reflecting their sequence similarity. In addition, the *A. thaliana* isoenzyme AT3G58750.1 grouped with several soursop isoenzymes, whereas the cherimoya isoenzymes Anche102Ch1g0031800.1 and Anche102Chr1g0031710.1 clustered together with one soursop isoenzyme and two *A. thaliana* sequences, as shown in Figure 4.

#### 2.3.2. Phylogenetic Analysis of MDH

**Figure 5:**
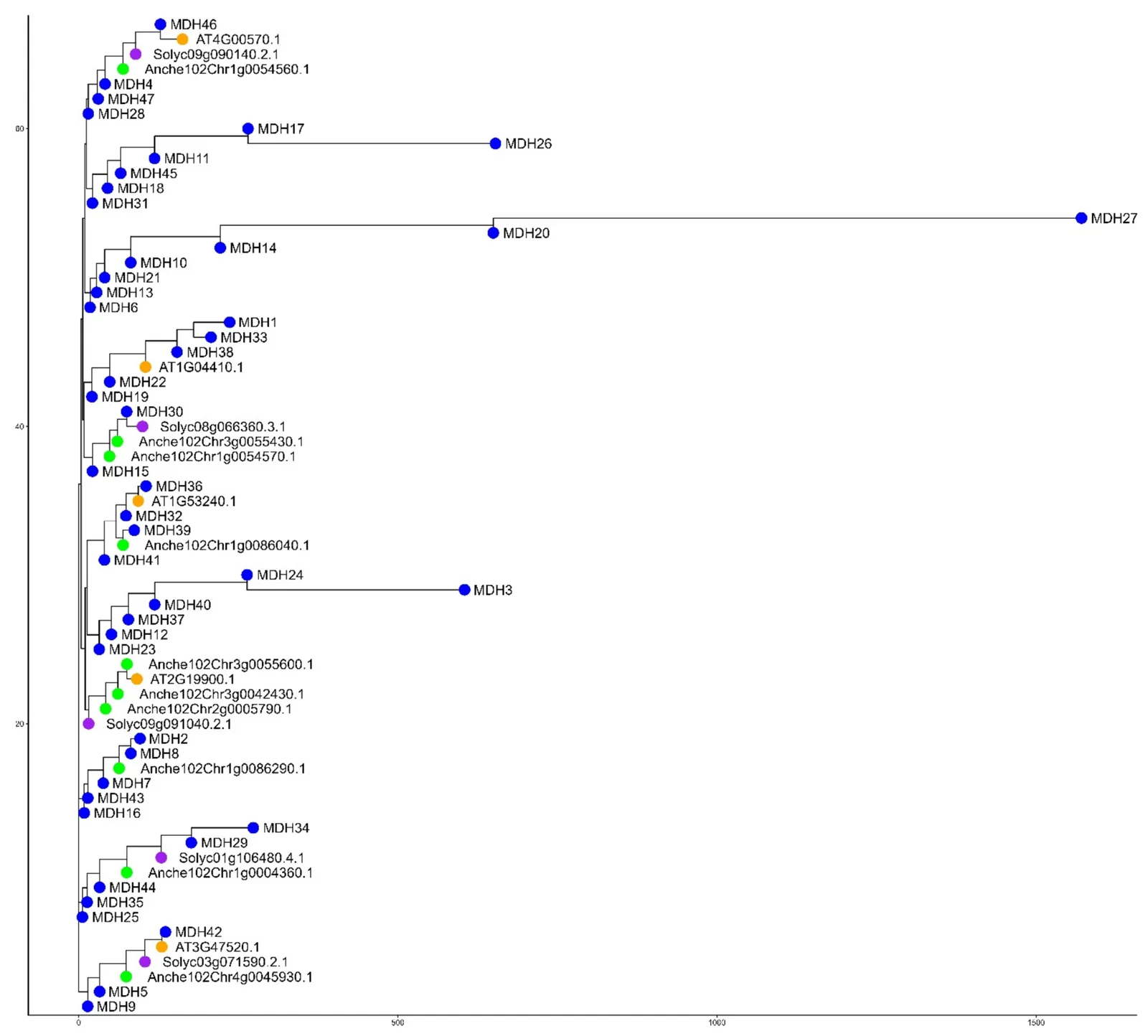
Neighbor-Joining phylogenetic tree of genes encoding the MDH enzyme. Blue corresponds to soursop, green to cherimoya, orange to *A. thaliana*, and purple to tomato. The values at the bottom (*x*-axis) represent the estimated genetic distance (branch length) derived from the multiple sequence alignment.

A total of 67 dehydrogenase-related protein sequences were identified in soursop, cherimoya, tomato, and Arabidopsis, containing the PF00180 and PF00056 domains, of which 47 corresponded to soursop fruit sequences (Figure 5). Functional annotation revealed that only *MDH3* corresponded to malate/lactate dehydrogenase, whereas *MDH1* and *MDH2* were annotated as isocitrate/isopropylmalate dehydrogenases. Accordingly, *MDH1* and *MDH2* were interpreted as dehydrogenases associated with organic acid metabolism rather than as malate dehydrogenases, whereas *MDH3* represents the candidate more directly associated with malate dehydrogenase activity. The Neighbor-Joining tree grouped the soursop isoenzymes (blue) into several clusters. For example, *MDH46, MDH11, MDH45, MDH18*, and *MDH31* were grouped within the same cluster, whereas *MDH26* was located in a neighboring branch. Similarly, *MDH6*, *MDH10*, *MDH21*, and *MDH13* formed another cluster. *MDH3*, *MDH24*, *MDH40*, *MDH37*, *MDH12*, and *MDH23* were also grouped together. The remaining soursop isoenzymes were distributed among clusters containing homologous sequences from tomato (purple), cherimoya (green), and *A. thaliana* (orange).

#### 2.3.3. Differential Gene Expression Analysis of CS and MDH Genes

**Figure 6:**
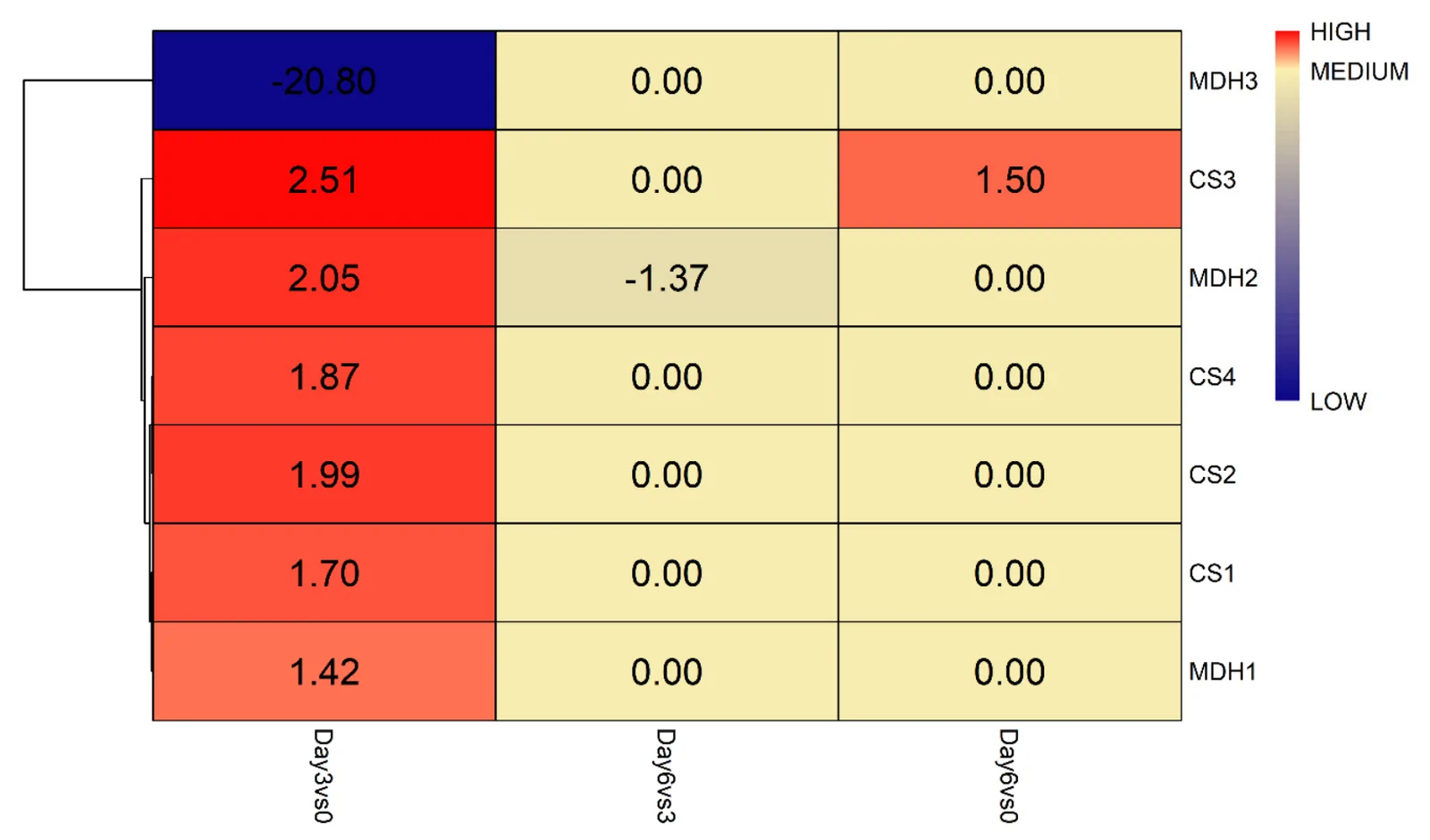
Heatmap of differentially expressed genes encoding MDH and CS enzymes showing log2FC values obtained from pairwise differential expression analyses (day 3 vs. day 0, day 6 vs. day 0, and day 6 vs. day 3). Colors represent gene expression levels. The *x*-axis corresponds to comparisons among storage days, and the *y*-axis shows differentially expressed isoenzymes with Log2FoldChange > 1 and adjusted *p*-value < 0.05.

Seven differentially expressed genes were identified, including four encoding CS (*CS1*, *CS2*, *CS3*, and *CS4*) and three encoding MDH (*MDH1*, *MDH2*, and *MDH3*). Based on PFAM domain annotation, *MDH1* and *MDH2* corresponded to isocitrate/isopropylmalate dehydrogenase, whereas *MDH3* was associated with malate/lactate dehydrogenase isoenzymes (Figure 6). It was observed that *CS2*, *CS4*, *CS1*, and *MDH2* were upregulated on day 3 compared with day 0 of postharvest storage.

The *CS3* gene showed increased expression in the day 3 vs. day 0 comparison (log2FC = 2.51). No differential expression was detected in the day 6 vs. day 3 comparison, whereas the gene remained upregulated in the day 6 vs. day 0 comparison (log2FC = 1.50). *MDH1* was upregulated in the day 3 vs. day 0 comparison (log2FC = 2.05) and downregulated in the day 6 vs. day 3 comparison (log2FC = −1.37), while no differential expression was detected in the day 6 vs. day 0 comparison. In contrast, *MDH3* was identified as a significantly downregulated gene in the day 3 vs. day 0 comparison (log2FC = −20.80), whereas no differential expression was detected in the remaining pairwise comparisons. The complete DESeq2 statistical parameters (baseMean, log2FoldChange, lfcSE, Wald statistic, *p*-value, and adjusted *p*-value) for the differentially expressed genes identified in the day 3 vs. day 0 comparison are provided in Table S1. Hierarchical clustering separated *MDH3* from the other genes according to its differential expression profile, while *CS1*, *CS2*, *CS3*, *CS4*, *MDH1*, and *MDH2* clustered together, reflecting similar expression patterns across the evaluated comparisons (Figure 6).

#### 2.3.4. Functional Enrichment Analysis

**Figure 7:**
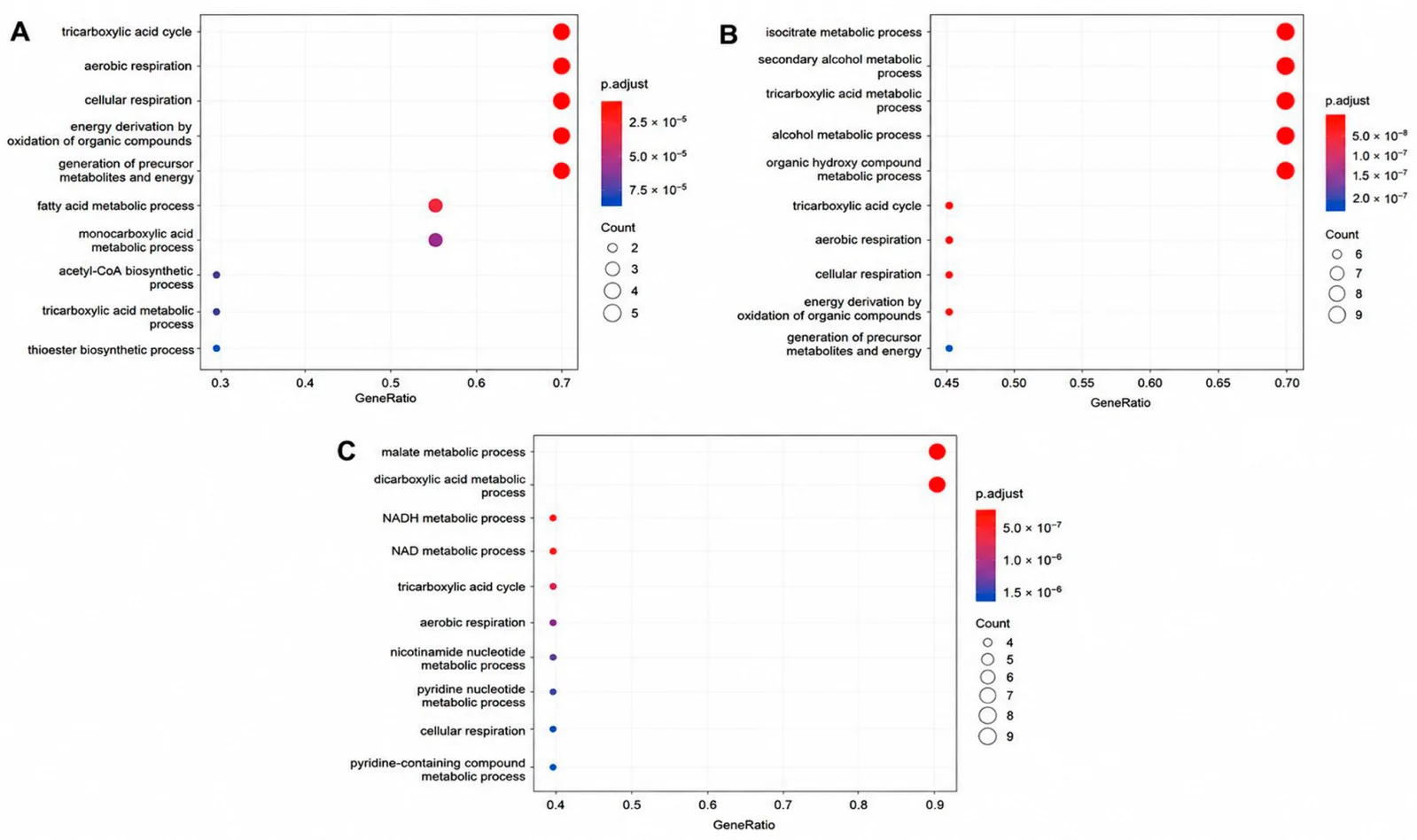
Functional enrichment analysis of citrate synthase (**A**), isocitrate/isopropylmalate dehydrogenase (**B**), and malate/lactate dehydrogenase (**C**). The bubble plot shows significantly enriched biological processes according to Gene Ontology (GO) analysis. The *y*-axis represents GO terms related to metabolic and energy processes, while the *x*-axis indicates the GeneRatio, corresponding to the proportion of genes associated with each term relative to the total number of analyzed genes.

Citrate synthase isoenzymes (Figure 7A), containing the PF00285 protein domain, showed functional enrichment mainly associated with the tricarboxylic acid (TCA) cycle, aerobic respiration, and cellular respiration. These enzymes are also involved in the generation of metabolic and energetic precursors, as well as in energy production through the oxidation of organic compounds. To a lesser extent, these isoenzymes were associated with fatty acid metabolism and monocarboxylic acid metabolism. On the other hand, isocitrate/isopropylmalate dehydrogenase isoenzymes containing the PF00180 domain (Figure 7B) were primarily related to isocitrate metabolism and the TCA cycle, sharing metabolic participation with citrate synthase isoenzymes within the same energy pathway. They were also associated with the generation of metabolic and energetic precursors in the cytosol, as well as with alcohol metabolic processes and the metabolism of hydrophilic organic compounds. However, they showed a weaker association with aerobic respiration and cellular respiration processes. Similarly, isoenzymes associated with the PF00056 domain (Figure 7C) were mainly related to malate metabolism and dicarboxylic acid metabolism. In addition, these isoenzymes were also associated with the TCA cycle, sharing participation in this metabolic pathway with the previously described enzymes. However, they showed a lower functional association with NAD and NADH metabolism, nicotinamide and pyridine nucleotide metabolism, as well as with aerobic and cellular respiration processes.

### 2.4. Gene Expression of CS and MDH

**Figure 8:**
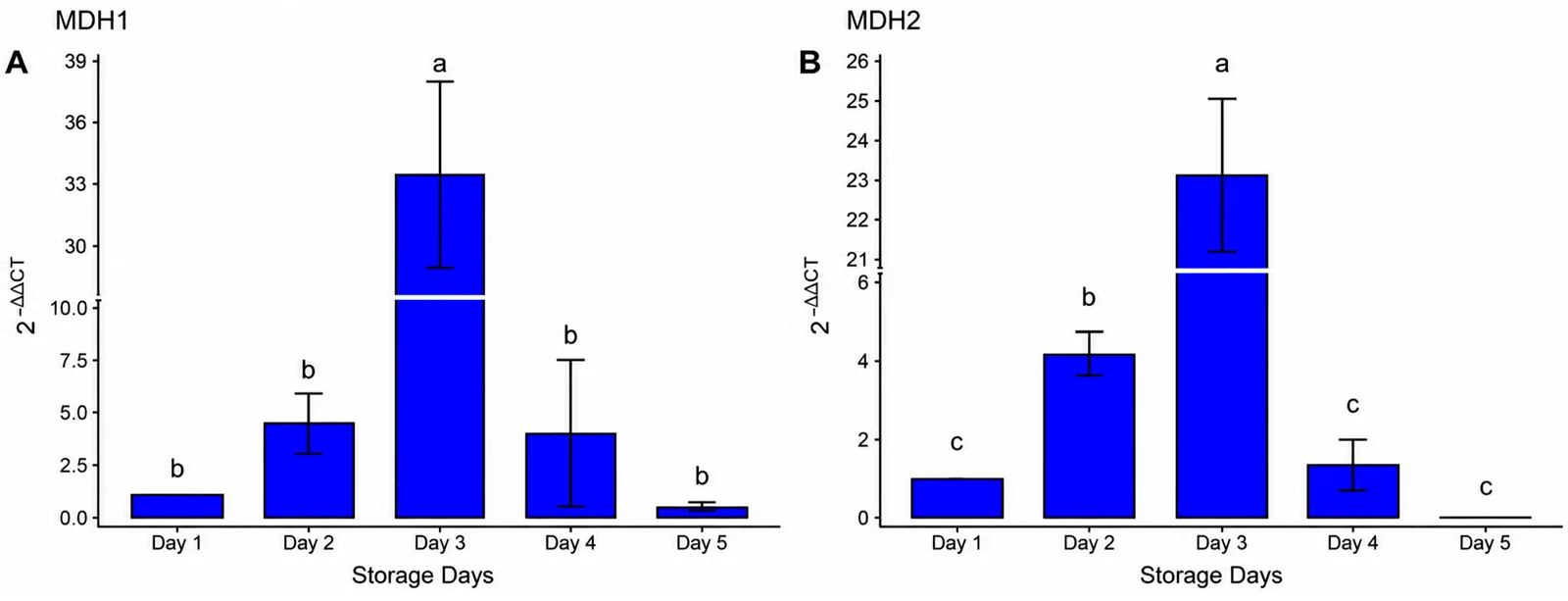
Relative expression of *MDH1* (**A**) and *MDH2* (**B**) genes in GUANAY-1 soursop fruits during storage at 26 ± 1 °C. The *x*-axis represents storage days, and the *y*-axis indicates relative expression levels. Different letters indicate significant differences among storage days according to Tukey’s HSD test (*p* < 0.05). Vertical error bars represent the standard deviation of the mean. The horizontal blank space indicates a break in the *y*-axis, which was used to facilitate data visualization due to the wide range of expression values.

The relative expression analysis showed significant differences in the expression levels of *MDH1* and *MDH2* (isocitrate/isopropylmalate dehydrogenase-related genes) throughout postharvest storage (*p* < 0.05), as shown in Figure 8. The highest expression level of *MDH1* was observed on day 3, with a value 33.40-fold higher than that recorded on day 1 (*p* < 0.05). Subsequently, expression significantly decreased, reaching relative values of 3.9 and 0.3 on days 4 and 5, respectively (Figure 8A). Similarly, *MDH2* showed significant differences in relative expression during storage (*p* < 0.05). The maximum expression level was observed on day 3, with a 23.11-fold increase compared to day 1. Thereafter, expression progressively decreased, reaching a 1.34-fold relative value on day 4 and returning to basal levels on day 5 (Figure 8B).

**Figure 9:**
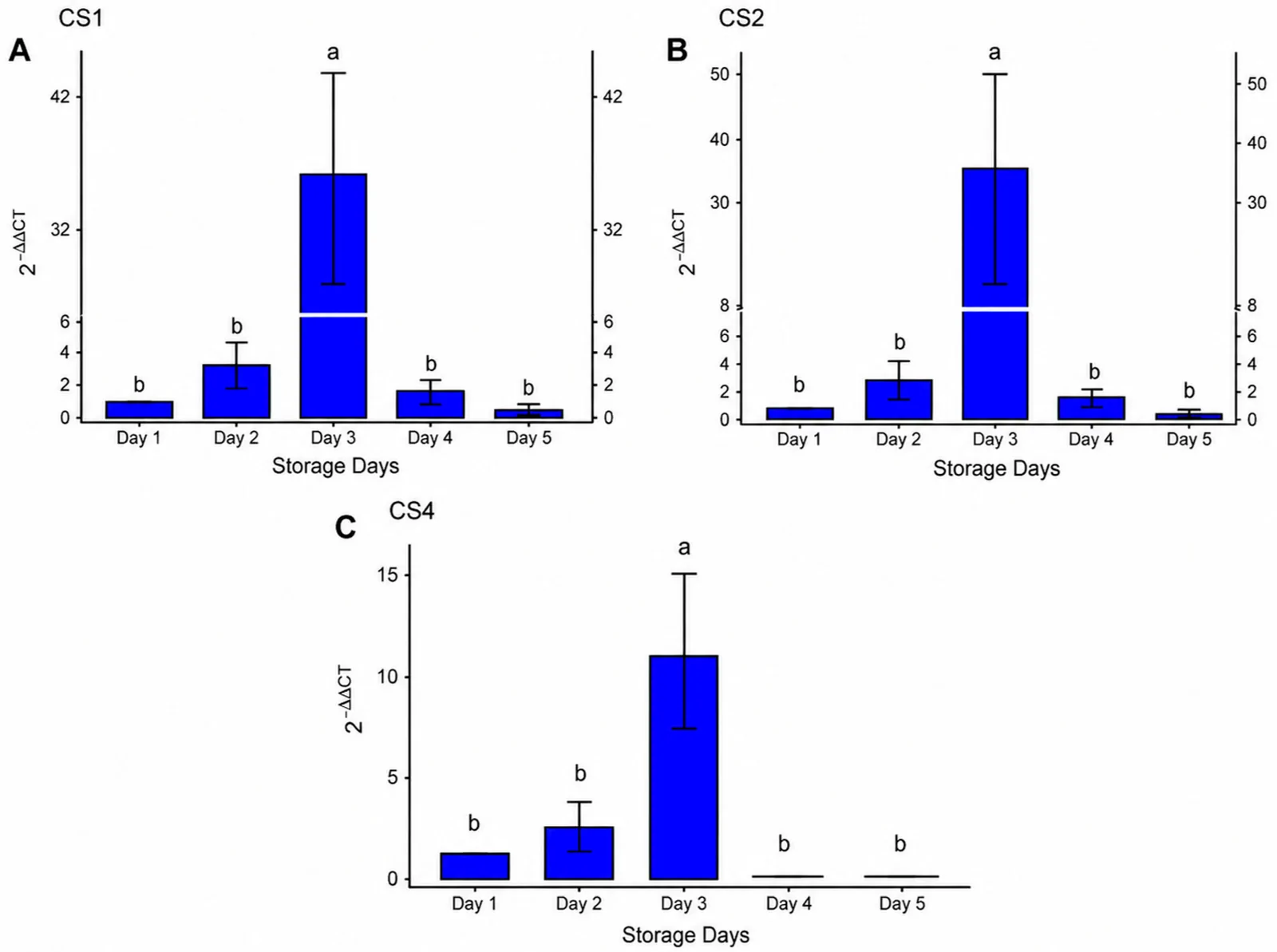
Relative expression of *CS1* (**A**), *CS2* (**B**), and *CS4* (**C**) genes in GUANAY-1 soursop fruits during storage at 26 ± 1 °C. The *x*-axis represents storage days, and the *y*-axis indicates relative expression levels. Different letters indicate significant differences among storage days according to Tukey’s HSD test (*p* < 0.05). Vertical error bars represent the standard deviation of the mean. The horizontal blank space indicates a break in the *y*-axis, which was used to facilitate data visualization due to the wide range of expression values.

The genes *CS1*, *CS2*, and *CS4* showed similar expression patterns during postharvest storage (Figure 9A–C). Significant differences were observed in their expression levels throughout storage (*p* < 0.05). Figure 9A shows the relative expression pattern of *CS1*, which increased progressively until reaching a maximum on day 3 (consumption ripening stage), with a 35.11-fold increase compared to day 1 (physiological maturity). This was followed by a decrease on day 4, where expression reached 1.58-fold, and on day 5 the gene showed a relative expression of 0.56-fold. Regarding *CS2* (Figure 9B), significant differences in relative expression were also observed during storage (*p* < 0.05). The highest expression level was recorded on day 3, with a 13.04-fold increase compared to day 1. Subsequently, expression steadily decreased, reaching a 1.77-fold relative value on day 4 and returning to basal levels on day 5. In contrast, *CS4* (Figure 9C) showed a progressive increase during the first three days of storage, reaching its maximum expression on day 3, with an 11.10-fold increase compared to day 1 (*p* < 0.05), while on days 4 and 5 the gene was not expressed.

### 2.5. Correlation Analysis

**Figure 10:**
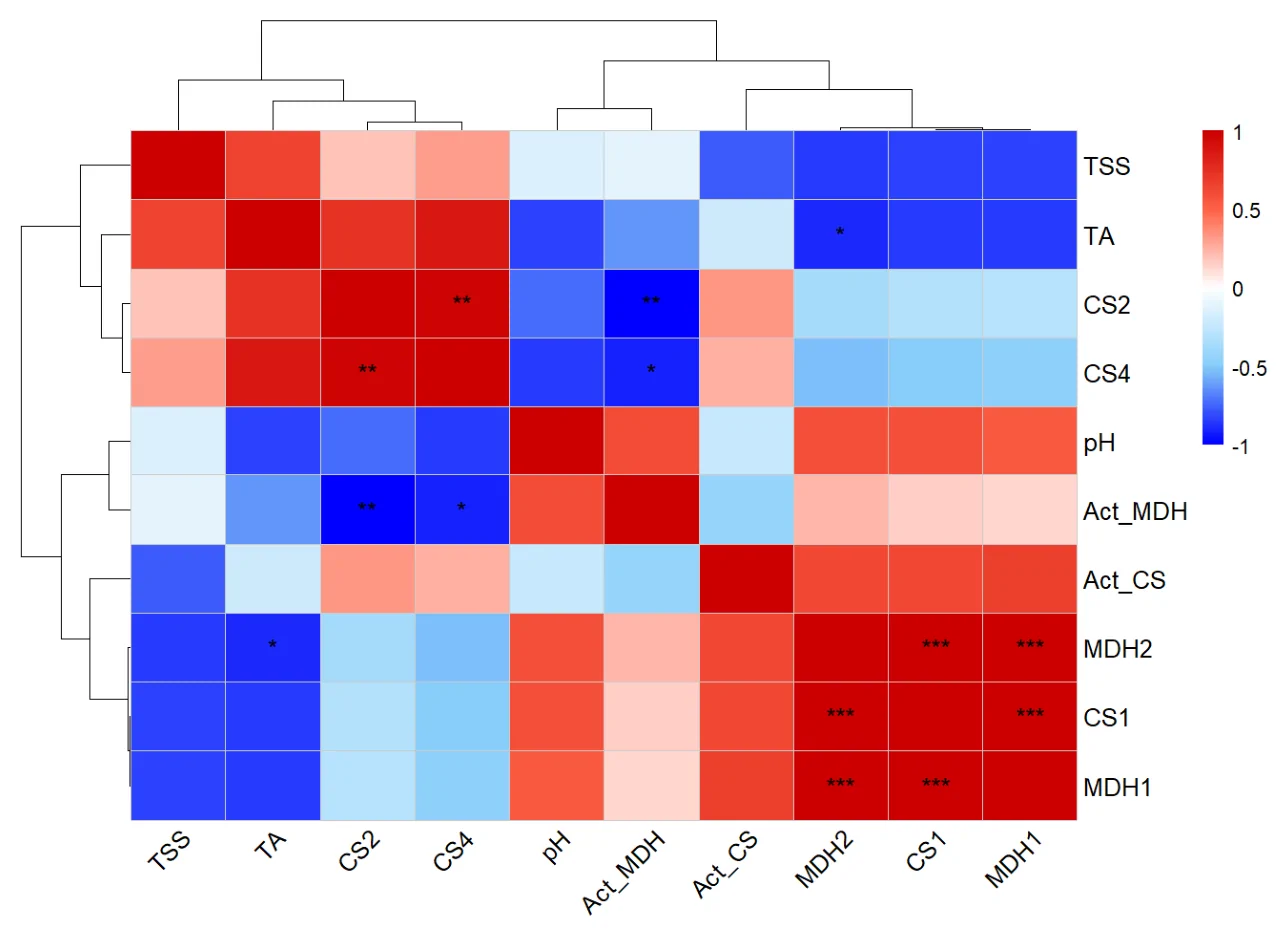
Pearson correlation analysis among physicochemical variables, enzymatic activities, and gene expression levels during soursop fruit ripening. Colors represent the strength and direction of correlations, ranging from negative (blue) to positive (red). Hierarchical clustering allowed for the identification of groups of variables with similar behavioral patterns. Act_CS indicates citrate synthase activity, and Act_MDH indicates malate dehydrogenase activity. Statistical significance levels are indicated by asterisks (* *p* < 0.05, ** *p* < 0.01, and *** *p* < 0.001).

The Pearson correlation analysis and hierarchical clustering were performed to explore overall temporal associations among physicochemical parameters, enzyme activities, and gene expression profiles during postharvest storage (Figure 10). Two major clusters were identified based on similarities in the overall variation patterns of the evaluated variables. The first cluster included TSS, TA, and the *CS2* and *CS4* genes, whereas the second cluster comprised pH, enzyme activities (Act_MDH and Act_CS), and the expression levels of *MDH1*, *MDH2*, and *CS1*. Positive and negative correlation coefficients indicate that the corresponding variables tended to vary in similar or opposite directions, respectively, across the complete set of biological observations during storage. Because these correlations summarize the overall variation across all storage days rather than day-to-day comparisons, they should be interpreted as exploratory temporal associations rather than evidence of direct biological or regulatory relationships.

## 3. Discussion

The present study integrated physicochemical, enzymatic, and gene expression analyses in soursop fruits during postharvest storage (days 1–5), revealing characteristic metabolic changes associated with ripening. The physicochemical values obtained at the consumption ripening stage (titratable acidity of 0.3% expressed as citric acid, pH > 5, and 3 °Brix) differed from those previously reported for soursop, where values of 1.11% and 0.6% citric acid [3,18], pH values between 4.5 and 5 [4,19], and total soluble solids (TSS) values of 12.88 and 9.87 °Brix [20,21] have been reported at the consumption ripening stage.

Although the temporal patterns of TSS and pH differ from the continuous trends commonly reported for many climacteric fruits, the values reported here are similar to those reported in the GUANAY-1 cultivar under similar postharvest storage conditions [15,16,17], supporting the hypothesis that these fluctuations may represent cultivar-specific physiological responses. This consistency across independent studies suggests that the observed patterns are reproducible under the evaluated conditions and are therefore unlikely to be solely attributable to analytical or sampling variability.

Soursop ripening involves dynamic changes in carbohydrate metabolism, respiration, and organic acid turnover. Accordingly, soluble sugars and organic acids are continuously synthesized, consumed, and redistributed during postharvest storage rather than changing monotonically. Such metabolic reprogramming may result in transient decreases and subsequent increases in physicochemical parameters, particularly in cultivars exhibiting complex postharvest physiology [12]. In climacteric fruits, these processes are tightly regulated by transcriptional networks and ethylene signaling, promoting metabolic reprogramming and contributing to changes in fruit quality attributes [22].

Although the observed fluctuations appear to be reproducible in the GUANAY-1 cultivar, differences among studies may also reflect variation in pre- and postharvest conditions, including edaphoclimatic factors. Wu et al. [23] reported that environmental conditions, such as soil pH, may influence fruit acidity and quality [23,24]. Therefore, the non-monotonic changes observed in TSS and pH are likely to reflect the postharvest physiological behavior of the GUANAY-1 cultivar under the evaluated storage conditions.

At the enzymatic level, changes in citrate synthase (CS) and malate dehydrogenase (MDH) activities may be related to modifications in energy metabolism during fruit ripening. These changes could be associated with the availability of substrates and cofactors, such as NADH, which modulate the activity of enzymes involved in energy metabolism, leading to physiological adjustments [25]. In particular, the increase observed on day 3 suggests the activation of a transitional metabolic phase.

On the other hand, phylogenetic and differential expression analyses showed that, among the 31 CS gene-encoding isoenzymes identified in soursop, four were differentially expressed and clustered within the same clade. Similarly, among the 67 MDH gene-encoding isoenzymes identified in soursop, three genes were differentially expressed. These clustered within clades together with isoenzymes from cherimoya and tomato, reflecting their evolutionary conservation and functional homology [26,27]. Likewise, GO annotation indicated that the identified DEGs were associated with biological processes related to the Krebs cycle. These findings are consistent with the participation of genes associated with energy metabolism during soursop fruit ripening.

Likewise, these genes showed correlations with physicochemical and biochemical variables, including pH, TSS, TA, citrate synthase activity (Act_CS), and malate dehydrogenase activity (Act_MDH). Because these associations were obtained from an exploratory correlation analysis performed during successive stages of postharvest storage, they should be interpreted as temporal associations rather than evidence of direct regulatory relationships.

In this context, the highest CS enzymatic activity was observed on day 3 (110.36 U mg^−1^ protein), together with the highest expression levels of *CS1*, *CS2*, *CS4*, *MDH1*, and *MDH2* (*p* < 0.05). In contrast, MDH showed its highest activity on day 2 (80.02 U mg^−1^ protein), followed by a relatively high activity on day 3 (48 U mg^−1^ protein). The increased expression of *CS1*, *CS2*, *CS4*, *MDH1*, and *MDH2* genes, together with the increase in citrate synthase activity on the third day of storage, may reflect a metabolic adjustment associated with the transition toward the consumption ripening stage. Since citrate synthase catalyzes the first irreversible step of the tricarboxylic acid cycle, the increase in its activity may indicate a greater involvement of this metabolic pathway during this stage. In climacteric fruits, activation of the Krebs cycle has been associated with the characteristic respiratory increase during ripening and with the supply of energy and intermediates required for various biosynthetic processes [14]. However, these interpretations are based on the enzymatic activity, gene expression patterns, and physicochemical parameters evaluated in this study, since individual organic acids, such as citrate and malate, were not directly quantified. Therefore, the proposed changes in the metabolism of these acids should be considered physiological inferences supported by the literature.

In contrast, the highest MDH activity was recorded one day earlier than that of CS, suggesting that malate metabolism may be activated early to maintain cellular redox balance through the reversible interconversion between malate and oxaloacetate, as well as to ensure the availability of oxaloacetate as a substrate for condensation with acetyl-CoA catalyzed by citrate synthase. This temporal sequence may be consistent with the hierarchical organization of organic acid metabolism described in fleshy fruits, where variations in malate and citrate levels reflect dynamic adjustments in Krebs cycle flux as energy and biosynthetic demands change during ripening [12,14].

Lagreze-Pérez [28] reported elevated CS activity during the consumption ripening stage in cherimoya (cv. Concha Lisa). Furthermore, González-Agüero et al. [29] analyzed the expression of genes encoding CS and MDH during the ripening of cherimoya fruits (cv. Concha Lisa) stored at 20 °C using qRT-PCR. The authors showed that the *Ac-cyNAD-MDH* gene exhibited a 4.0-fold increase in expression on day 2, whereas the *AcNAD-SE* gene showed a twofold increase during the same period. Likewise, the *Ac-mCS* gene showed an approximately 1.8-fold increase compared with day 1. In contrast, in the present study, the candidate CS- and MDH-related genes reached their highest expression on day 3 of storage, suggesting differences in ripening behavior between species or cultivation conditions.

Previous studies indicate that ripening is a coordinated process involving the activation of gene modules related to respiration, carbon metabolism, and energy production [5,28]. In climacteric fruits, ethylene acts as a key phytohormone that regulates the expression of genes associated with respiration, cell wall degradation, and the metabolism of sugars and organic acids [7,30,31]. In the GUANAY-1 variety, peak ethylene production has been reported on days 3 and 5 of storage [19]. The first peak of ethylene occurred close to the highest expression levels of the candidate CS- and MDH-related genes observed on day 3 of storage. This suggests a possible temporal association between ethylene production and gene expression during ripening. However, further studies are required to confirm this hypothesis.

A limitation of the present study is that only a limited number of candidate genes and enzymes were evaluated during a five-day postharvest storage period. In addition, individual organic acids, such as citrate and malate, were not directly quantified; therefore, the proposed changes in organic acid metabolism are inferred from enzyme activities, gene expression, and physicochemical parameters.

Overall, the results identify day 3 of storage (consumption ripening stage) as a key transition point during soursop fruit ripening, characterized by increased citrate synthase activity together with the highest expression levels of several candidate CS- and MDH-related genes. These observations support the hypothesis that this stage is associated with important metabolic changes during ripening and provide a foundation for future studies addressing the regulation of energy metabolism during postharvest fruit development.

## 4. Materials and Methods

### 4.1. Plant Material

Soursop (*A. muricata*) GUANAY-1 fruits were harvested at physiological maturity (approximately 150–160 days after anthesis). Fruits free of visible symptoms of disease and mechanical damage were selected based on their shape, size, and peel color, as previously described [32]. The fruits were collected from a 28-year-old orchard located in Venustiano Carranza, Nayarit, Mexico (21°3′22.77″ N, 104°58′39.73″ W). Immediately after harvest, the fruits were transported, disinfected with 2.0% sodium hypochlorite, and rinsed with distilled water. Subsequently, they were stored at 26 ± 1 °C and an average relative humidity of 66%. Three fruits were sampled on each storage day over a five-day period (days 1, 2, 3, 4, and 5). Day 1 corresponded to the physiological maturity stage, day 3 to the consumption ripening stage, and day 5 to the onset of senescence. Stage classification was based on the characteristic external and internal attributes previously established for the GUANAY-1 cultivar, particularly peel color and pulp texture, following the criteria described by Díaz-Rincón et al. [4] and Palomino-Hermosillo et al. [15].

Approximately 100 g of mesocarp tissue was collected from randomly selected regions of each fruit. One portion of the mesocarp was used immediately for physicochemical analyses, whereas the remaining tissue was ground in liquid nitrogen and stored at −20 °C for enzyme activity assays. In addition, approximately 50 g of mesocarp tissue was collected from each fruit, preserved in RNAlater^®^ solution, and stored at −20 °C. The samples were subsequently ground in liquid nitrogen and stored at −80 °C until RNA extraction.

### 4.2. Physicochemical Analysis

Fruit juice was extracted from the soursop mesocarp and used for the determination of pH, total soluble solids (TSS), and titratable acidity (TA). The pH was measured using a digital pH meter (HI2210, Hanna Instruments, Woonsocket, RI, USA). Total soluble solids (TSS) were determined using a digital refractometer (HI96801, Hanna Instruments, Woonsocket, RI, USA), and the results were expressed as °Brix. Titratable acidity (TA) was determined by volumetric titration according to the AOAC methodology using phenolphthalein as the indicator and 0.01 N NaOH as the titrant.

### 4.3. Enzyme Extraction

The enzyme extract was prepared according to the method described by Gonzalez-Agüero et al. [29] with minor modifications. Briefly, 600 mg of powdered plant tissue was homogenized with 1 mL of extraction buffer containing 0.2 M Tris-HCl (pH 8.2), 0.6 M sucrose, and 10 mM isoascorbic acid. The homogenate was transferred to 2 mL microcentrifuge tubes and centrifuged at 10,000× *g* for 4 min at 4 °C using a refrigerated centrifuge (D-37520, Sigma-Aldrich^®^, St. Louis, MO, USA). The resulting supernatant was centrifuged again at 10,000× *g* for 10 min at 4 °C. Subsequently, an 800 µL aliquot of the supernatant was mixed with 200 µL of grinding buffer and 1 mL of extraction buffer containing 0.2 M Tris-HCl (pH 8.2), 10 mM isoascorbic acid, and 0.1% (*v*/*v*) Triton X-100 to preserve the sample and prevent tissue oxidation. The mixture was centrifuged at 15,000× *g* for 15 min at 4 °C. The supernatant was discarded, and the pellet was resuspended in 1.2 mL of extraction buffer. A 400 µL aliquot of the suspension was designated as Tube A, whereas the remaining 800 µL (Tube B) was first dialyzed overnight (25–27 °C) using dialysis tubing (55 mm; molecular weight cut-off: 8–14 kDa) against 0.2 M Tris-HCl containing 10 mM isoascorbic acid and subsequently used for citrate synthase activity determination.

### 4.4. Enzyme Activity Assays

Enzyme activities were determined according to the method described by González-Agüero et al. [29]. Reaction mixtures were prepared for each enzyme in a final volume of 250 µL as follows:

Citrate synthase (CS): 40 mM Tris-HCl (pH 9.0), 40 µM 5,5′-dithiobis-(2-nitrobenzoic acid) (DTNB), 40 µM acetyl-CoA, 4 mM oxaloacetate, and 70 µL of Tube B.

Malate dehydrogenase (MDH): 40 mM Tris-HCl (pH 8.2), 2 mM MgCl_2_, 10 mM KHCO_3_, 0.5 mM reduced glutathione (GSH), 2 mM oxaloacetate, 150 µM NADH, and 94 µL of Tube A.

Citrate synthase activity was measured at 412 nm, whereas malate dehydrogenase activity was measured at 340 nm using a Multiskan™ GO microplate spectrophotometer (Thermo Fisher Scientific, Waltham, MA, USA). Absorbance readings were recorded every 30 s for 5 min and absorbance was recorded immediately using a Multiskan GO microplate spectrophotometer (Thermo Fisher Scientific, Vantaa, Finland). Absorbance values were obtained directly using SkanIt software 4.1 without applying path-length correction. Reaction blanks without enzyme extract were included for background correction. Enzyme activity was calculated based on the change in absorbance per minute (ΔA/min) using the molar extinction coefficients of DTNB (13.6 mM^−1^ cm^−1^) for CS and NADH (6.22 mM^−1^ cm^−1^) for MDH. Enzyme activity was expressed as U mg^−1^ protein, where one unit (U) corresponds to the amount of enzyme catalyzing the conversion of 1 µmol of substrate per minute. Total protein concentration in Tubes A and B was determined spectrophotometrically according to the method of Bradford [33].

### 4.5. Bioinformatic Analysis

#### 4.5.1. Phylogenetic Analysis

Soursop (*A. muricata*), tomato (*Solanum lycopersicum*), *A. thaliana*, and cherimoya (*Annona cherimola*) sequences containing the PFAM protein domains PF00285, PF00180, and PF00056, associated with citrate synthase (CS) and malate dehydrogenase (MDH), were identified. For soursop, transcriptome data from BioProject PRJNA804904 and the Annomics Database (http://perseo.uan.mx/bioinformatica/annomicsdatabase; accessed on 20 March 2025) were used. Cherimoya sequences were retrieved from the IHSM Subtropicals database (https://ihsmsubtropicals.uma.es/easy_gdb/tools/search/search_input.php; accessed on 20 March 2025). Tomato and *A. thaliana* sequences were obtained from the Phytozome database (https://phytozome-next.jgi.doe.gov/; accessed on 20 March 2025).

The sequences were concatenated into a FASTA file and aligned using the Clustal Omega algorithm implemented in R v4.4.3 with the Biostrings package. A phylogenetic tree was subsequently constructed using the Neighbor-Joining method [34] with default parameters. Bootstrap support values were not calculated because the analysis was intended to provide an exploratory assessment of the relationships among the identified sequences and their homologs. All analyses were performed in R v4.4.3 using the Biostrings, msa, phangorn, ggtree, ggplot2, ape, and dplyr packages.

#### 4.5.2. Differential Gene Expression Analysis

Differential gene expression analysis was performed using the RSEM count matrix obtained from a previously published RNA-seq transcriptome of the GUANAY-1 soursop variety (BioProject PRJNA804904) [15]. The transcriptomic dataset was generated using three biological replicates sampled at physiological maturity (day 0), consumption ripening (day 3), and the onset of senescence (day 6) during storage at 28 ± 2 °C. Pairwise comparisons (day 3 vs. day 0, day 6 vs. day 3 and day 6 vs. day 0) were analyzed using the DESeq2 package in R v4.4.3 [35]. Genes with an absolute log2 fold change (|Log2FC| > 1) and an adjusted *p*-value ≤ 0.05 were considered significantly differentially expressed. A heatmap of the DEGs was generated in R using the ggplot2 and pheatmap packages. The differentially expressed CS and MDH genes identified from this transcriptomic analysis were subsequently selected for independent validation by qRT-PCR using a separate postharvest experiment conducted under the conditions of the present study (26 ± 1 °C), in which fruits were sampled daily (days 1–5) to obtain higher temporal resolution of gene expression during ripening.

#### 4.5.3. Functional Enrichment Analysis

Gene Ontology (GO) annotations for the identified DEGs were obtained using the trinotateR and biomaRt packages in R, following the methodology described by Díaz-Rincón et al. [36]. Because soursop is a non-model species with limited genomic resources, functional annotation was performed using *A. thaliana* orthologs as the reference. Subsequently, the Database for Annotation, Visualization and Integrated Discovery (DAVID) platform (https://davidbioinformatics.nih.gov/; accessed on 12 April 2025) was used to identify the biological processes and metabolic pathways associated with the DEGs.

Enrichment analysis was performed using the annotated *A. thaliana* gene set available in DAVID as the background gene set and the modified Fisher’s exact test (EASE score). *p*-values were adjusted for multiple testing using the Benjamini–Hochberg false discovery rate (FDR) method, and terms with an adjusted *p* < 0.05 were considered statistically significant.

Because the analysis was performed on a pre-selected set of candidate genes, the GO enrichment analysis was considered descriptive and exploratory, serving primarily to confirm the expected functional annotation of the identified DEGs.

### 4.6. Primer Design

Primers were designed from the sequences of the differentially expressed genes using the online PrimerQuest Tool (Integrated DNA Technologies, IDT). Primer design parameters included an annealing temperature ranging from 55 to 63 °C, a GC content of approximately 50%, a primer length of 18–25 nucleotides, and an expected amplicon size of 75–130 bp.

Primer specificity was evaluated using Primer-BLAST at the National Center for Biotechnology Information (NCBI) (http://www.ncbi.nlm.nih.gov/tools/primer-blast/; accessed on 25 April 2025). Primer identifiers, sequences, and expected amplicon sizes are listed in Table 1.

### 4.7. RNA Extraction and cDNA Synthesis

Total RNA was extracted according to the method described by Gudenschwager et al. [37] with minor modifications. Briefly, 100 mg of powdered tissue was homogenized in 1 mL of lysis buffer containing 200 mM sodium borate decahydrate (pH 9.0), 30 mM EGTA, 1% (*w*/*v*) SDS, 1% (*w*/*v*) sodium deoxycholate, 0.5% (*v*/*v*) Triton X-100, and 2% (*v*/*v*) β-mercaptoethanol. The homogenate was subsequently precipitated with 80 μL of 4 M LiCl overnight. The samples were then centrifuged at 15,000× *g* for 40 min, and the resulting pellet was resuspended in 100 μL of diethyl pyrocarbonate (DEPC)-treated water and 10 μL of 3 M sodium acetate. Subsequently, 110 μL of chloroform:isoamyl alcohol (1:1, *v*/*v*) and DEPC-treated water were added. RNA was precipitated again by adding 50 μL of isopropanol and 12 μL of 3 M sodium acetate, followed by centrifugation under the same conditions. The RNA pellet was washed with 200 μL of 80% ethanol, resuspended in 50 μL of DEPC-treated water, and stored at −80 °C until further use. RNA concentration and purity were determined using a Multiskan™ GO microvolume spectrophotometer (Thermo Fisher Scientific, Waltham, MA, USA) by measuring the absorbance ratio at 260/280 nm. RNA integrity was assessed by electrophoresis on 1.0% agarose gels stained with 6 μL of SYBR™ Gold Nucleic Acid Gel Stain (Thermo Fisher Scientific). Electrophoresis was performed in 1× TAE (Tris-acetate-EDTA) buffer at a constant voltage of 80 V for 120 min. The gels were visualized using a benchtop UV transilluminator coupled to a PhotoDoc-IT System imaging system. First-strand cDNA was synthesized from 400 ng of total RNA using the SuperScript^®^ III First-Strand Synthesis System for RT-PCR (Invitrogen, Carlsbad, CA, USA) according to the manufacturer’s instructions. The synthesized cDNA was stored at −20 °C until further use.

### 4.8. Polymerase Chain Reaction (PCR)

PCR amplification was performed using a T100™ Thermal Cycler (Bio-Rad Laboratories, Hercules, CA, USA) with Green Taq Master Mix (Sigma-Aldrich, St. Louis, MO, USA). Each PCR reaction contained 0.5 μL of each forward and reverse primer (10 μM), 12 μL of Green Taq Master Mix, 1 μL of cDNA (30 ng), and 6 μL of nuclease-free water. The PCR cycling conditions consisted of an initial denaturation at 94 °C for 5 min, followed by 40 cycles of denaturation at 94 °C for 45 s, annealing at 60 °C for 30 s, and extension at 72 °C for 1 min, with a final extension at 72 °C for 10 min. PCR products were separated by electrophoresis on 1.0% agarose gels at 80 V for 1.5 h. The gels were visualized using a UV transilluminator coupled to a PhotoDoc-It gel documentation system (Ultra-Violet Products Ltd., Upland, CA, USA). Finally, the PCR products were sequenced by Macrogen Inc. (Seoul, Republic of Korea) to confirm their identity.

### 4.9. Quantitative Real-Time PCR (qRT-PCR)

Relative gene expression was quantified by quantitative real-time PCR (qRT-PCR) using a 7500 Fast Real-Time PCR System (Applied Biosystems, Foster City, CA, USA) and FastStart Universal SYBR Green Master Mix (Sigma-Aldrich, St. Louis, MO, USA). Each qRT-PCR reaction contained 0.5 μL of each forward and reverse primer (10 μM), 10 μL of FastStart Universal SYBR Green Master Mix, 2 μL of cDNA, and 7 μL of nuclease-free water, for a final reaction volume of 20 μL. Relative standard curves were generated using serial dilutions of cDNA to determine amplification efficiency, primer specificity, and the optimal cDNA concentration for amplification of the target genes. The qRT-PCR cycling conditions consisted of an initial denaturation at 94 °C for 5 min, followed by 38 cycles of denaturation at 94 °C for 45 s, annealing at 60 °C for 30 s, and extension at 72 °C for 30 s, with a final extension at 72 °C for 10 min. A melting curve analysis was performed at the end of each amplification run to verify primer specificity and the absence of nonspecific amplification products and primer–dimer formation. Two candidate reference genes, 18S rRNA (*18s*) and ubiquitin-conjugating enzyme (*UBCc*), were evaluated as internal controls for qRT-PCR normalization. Both reference genes showed comparable normalization trends across the evaluated storage stages. Considering these results and the previous validation reported by Berumen-Varela et al. [32], *18s* was selected as the internal reference, with day 1 used as the calibrator for the final relative expression analyses. Relative transcript abundance was calculated using the 2^−^^∆∆CT^ method [38].

### 4.10. Correlation Analysis

Pearson correlation analysis was performed using the mean technical replicate value obtained for each biological replicate. Three independent biological replicates were analyzed at each of the five storage days (15 observations per variable). Because the observations represent successive stages of fruit ripening, the analysis was intended to describe temporal associations among variables rather than infer causal or regulatory relationships. Subsequently, the correlation matrix was computed using the Pearson coefficient, and the results were visualized as a heatmap with hierarchical clustering using the pheatmap package in R. The color intensity of the cells represented the magnitude and direction of the correlations, while asterisks indicated statistically significant correlations (*p* < 0.05). Statistical significance is presented descriptively and should be interpreted with caution because no multiple testing correction was applied.

### 4.11. Statistical Design

A completely randomized design was used to analyze physicochemical parameters, enzymatic activities, and gene expression data. Data homoscedasticity and normality were assessed using Bartlett’s and Shapiro–Wilk tests, respectively. All data were analyzed using analysis of variance (ANOVA) with a significance level of *p* < 0.05. When ANOVA indicated significant differences, Tukey’s post hoc test was performed at a 5% significance level to compare means among storage days. Physicochemical parameters and enzymatic activities were determined using three biological replicates with six technical measurements. Gene expression analysis was performed using three biological replicates with three technical measurements. In all cases, one fruit was considered a biological replicate. All analyses were conducted in R using the packages readxl, tidyverse, agricolae, and multcompView.

## 5. Conclusions

The present study demonstrates that physicochemical changes, CS and MDH enzymatic activities, and the expression of selected *CS* and *MDH* genes vary throughout postharvest storage of GUANAY-1 soursop fruit. These findings reveal temporal associations among these variables during fruit ripening and provide a basis for future studies to better understand organic acid metabolism during postharvest ripening.

## Figures and Tables

**Table 1:** Primer sequences designed for the differentially expressed CS and MDH isoenzyme genes. ID: gene identifier; Sequence: primer nucleotide sequence, F means forward and R reverse; Length: primer length; GC (%), guanine–cytosine content; expected PCR product size (bp).

ID	Sequence	Length (bp)	GC %	Expected Amplicon Size (bp)
*MDH1*	F: CCACTGTACTCTCCCTCAGTAT	22	50	83
	R: ACCAGCCTCTCTGGCTATAA	20	50	
*MDH2*	F: GAATGACTGGCAGAAGCAAATC	22	45	98
	R: GTGGGTGTTCGTCCCATAAT	20	50	
*CS1*	F: TTCGCACCATCAGCATCTT	19	47	90
	R: GAACTGTCAGAGCTACCGATTT	22	45	
*CS2*	F: CGCTGCCTTCATACAGTAGTT	21	48	102
	R: CTTCTGGCGCAGTTGATTTG	20	50	
*CS4*	F: CAGATCCCTATGCTCCTTGAAAC	23	48	104
	R: GGCCTGTCAATGCATCTTCT	20	50	
*18s*	F: CCTTCGGGATCGGAGTAATG	20	55	137
	R: AGATACCGTCCTAGTCTCAACC	22	50	
*UBCc*	Fw: ACCTCTATCCAGTCTCTCCTC	21	52	128
	Rv: TGAGATAGTGGAGCAGAGCT	20	50	

## Data Availability

The original contributions presented in this study are included in the article/Supplementary Materials. Further inquiries can be directed to the corresponding author.

## Supplementary Materials

The following supporting information can be downloaded at: https://www.mdpi.com/article/10.3390/ijms27167174/s1.

## Abbreviations

The following abbreviations are used in this manuscript:

CS	Citrate Synthase
MDH	Malate Dehydrogenase
TSS	Total Soluble Solids
TA	Titratable Acidity
TCA	Tricarboxylic Acid Cycle
PEPC	Phosphoenolpyruvate Carboxylase
pH	Potential of Hydrogen
AOAC	Association of Official Analytical Collaboration
PFAM	Protein Families Database
RSEM	RNA-Seq by Expectation-Maximization
DEGs	Differentially Expressed Genes
GO	Gene Ontology
DAVID	Database for Annotation, Visualization and Integrated Discovery
IDT	Integrated DNA Technologies
NCBI	National Center for Biotechnology Information
GC	Guanine–Cytosine Content
EGTA	Ethylene Glycol-bis(β-aminoethyl Ether)-N,N,N′,N′-Tetraacetic Acid
SDS	Sodium Dodecyl Sulfate
DEPC	Diethyl Pyrocarbonate
RNA	Ribonucleic Acid
SYBR	SYBR Green
EDTA	Ethylenediaminetetraacetic Acid
cDNA	Complementary DNA
PCR	Polymerase Chain Reaction
qRT-PCR	Quantitative Real-Time Polymerase Chain Reaction
rRNA	Ribosomal Ribonucleic Acid
ANOVA	Analysis of Variance
NAD	Nicotinamide Adenine Dinucleotide
NADH	Reduced Nicotinamide Adenine Dinucleotide
Act_CS	Citrate Synthase Activity
Act_MDH	Malate Dehydrogenase Activity
