# Supplementary material for: Dynamics of Citrate Synthase and Malate Dehydrogenase Gene Expression, Enzymatic Activity, and Physicochemical Changes During Postharvest Storage of Soursop (Annona muricata L.) GUANAY-1 Fruit
Source: Int J Mol Sci. 2026 Aug 11;27(16):7174. doi: 10.3390/ijms27167174 (PMC13513042; doi:10.3390/ijms27167174)
Supplement: Supplementary file 1 [file ijms-27-07174-s001.zip › ijms-4454895-supplementary.pdf]

Table S1. DESeq2 statistical parameters for the differentially expressed genes (DEGs) analyzed in this study (Day 3 vs Day 0). The table includes the mean normalized expression level across all samples (baseMean), log2 fold change (log2FC), standard error of the log2 fold change (lfcSE), Wald statistic, raw *p*-value, Benjamini–Hochberg adjusted p-value (padj), PFAM annotation, Gene Ontology (GO) assignment, and direction of differential expression. Genes with |log2FC| > 1 and adjusted p-value ≤ 0.05 were considered differentially expressed.

Gene	Trinity ID	Functional annotation	PFAM	GO term	GO ontology	baseMean	log2FC	lfcSE	Wald statistic	p-value	padj	Regulation
**CS1**	TRINITY_DN12694_c1_g1	Citrate synthase, C-terminal domain	PF00285	GO:0004108	Molecular function	26.29	1.705	0.639	2.666	7.68×10^{-3}	2.92×10^{-2}	Up
**MDH2**	TRINITY_DN14064_c2_g2	Isocitrate/isopropylmalate dehydrogenase	PF00180	GO:0000287	Molecular function	40.24	2.052	0.535	3.834	1.26×10^{-4}	7.34×10^{-4}	Up
**MDH1**	TRINITY_DN14762_c4_g1	Isocitrate/isopropylmalate dehydrogenase	PF00180	GO:0000287	Molecular function	64.75	1.425	0.412	3.456	5.49×10^{-4}	2.82×10^{-3}	Up
**CS4**	TRINITY_DN15254_c4_g5	Citrate synthase, C-terminal domain	PF00285	GO:0004108	Molecular function	49.47	1.871	0.501	3.738	1.86×10^{-4}	1.05×10^{-3}	Up
**CS3**	TRINITY_DN15254_c4_g6	Citrate synthase, C-terminal domain	PF00285	GO:0004108	Molecular function	38.11	2.516	0.877	2.870	4.11×10^{-3}	2.13×10^{-2}	Up
**CS2**	TRINITY_DN15254_c4_g7	Citrate synthase, C-terminal domain	PF00285	GO:0004108	Molecular function	16.83	1.993	0.797	2.499	1.25×10^{-2}	4.40×10^{-2}	Up
**MDH3**	TRINITY_DN15355_c1_g1	Lactate/malate dehydrogenase, NAD-binding domain	PF00056	GO:0000746	Biological process	18.24	−20.796	4.410	−4.716	2.41×10^{-6}	1.78×10^{-5}	Down
